# Telemedicine Intensive Care Unit (Tele-ICU) Implementation During COVID-19: A Scoping Review

**DOI:** 10.7759/cureus.25133

**Published:** 2022-05-19

**Authors:** Shantele Kemp Van Ee, Heather McKelvey, Timothy Williams, Benjamin Shao, Wei-Ting Lin, Justin Luu, Divya Sunny, Shubhangi Kumar, Shreya Narayan, Alexandra Urdaneta, Luis Perez, Hailey Schwab, Sean Riegle, Robin J Jacobs

**Affiliations:** 1 Medicine, Nova Southeastern University Dr. Kiran C. Patel College Of Osteopathic Medicine, Fort Lauderdale, USA; 2 Medical and Behavioral Research, Health Informatics, Medical Education, Nova Southeastern University Dr. Kiran C. Patel College Of Osteopathic Medicine, Fort Lauderdale, USA

**Keywords:** pandemic, covid-19, intensive care, intensivist, critical care, telemedicine

## Abstract

Telemedicine intensive care unit (Tele-ICU) programs entail command centers staffed with intensivists and critical care nurses who electronically aid with and deliver real-time information to frontline clinicians. The benefits of Tele-ICU are numerous, but the barriers to it often prove insurmountable, accounting for slow adoption in rural and underserved areas where it is needed the most. Remote monitoring can quickly detect patient deterioration, while consultation provided by a remote intensivist expands the capabilities of smaller facilities. The emergence of the coronavirus disease 2019 (COVID-19) pandemic has brought about a sense of urgency, paving the way for the successful adaptation of tele-intensive care concepts. The goal of this scoping review is to map out the available published data regarding healthcare professionals’ experiences with implementing Tele-ICU modalities during the COVID-19 pandemic. A primary literature search was performed on PubMed/MEDLINE and the Cumulative Index to Nursing and Allied Health Literature (CINAHL) databases from October 2020 to October 2021. Of the 1,083 records screened, 19 were identified as meeting our inclusion criteria and selected for the final scoping review. Five major areas of Tele-ICU use were identified: teleconsultation, telerounding, telemonitoring, family visitation via teleconference, and changing of hospital infrastructure. A heterogeneous mix of improvised Tele-ICU platforms emerged with a common theme of interdisciplinary and family collaboration in the care of critically ill patients. Existing Tele-ICU systems were expanded, and novel programs were launched. A groundbreaking national network in the U.S. (NETCCN) will standardize the deployment of Tele-ICU and expand its reach. Future research should focus on determining accurate costs and the most reliable forms of remote communication, physician compact agreement licensure, the practical composition of Tele-ICU teams, and standardized access to the electronic health record.

## Introduction and background

While the utilization of telemedicine has progressed in recent years in all specialties to advance patient care and address staffing shortages, its adoption in critical care has moved at a slower pace due to variant methods of implementation, thereby yielding mixed outcomes [1]. A wide variety of telemedicine models have been introduced, including remote patient monitoring and intensivist-led consultation via teleconference with clinicians [2]. As necessity is the mother of invention, the burden imposed by the coronavirus disease 2019 (COVID-19) pandemic on the healthcare system has provided the impetus necessary to expand telemedicine for implementing impromptu changes in rapidly overburdened intensive care units (ICUs).

ICUs have actively utilized technology over the past several decades to improve compliance with best practices in managing a wide variety of critical care populations, both rural and urban [3]. In 2010, less than 8% of ICU beds utilized telemedicine in the United States; however, by 2018, that number grew to approximately 25% [4]. Remote monitoring and care have allowed for increased vigilance for patient deterioration while expanding the capacity of smaller units and decreasing staff burnout [5]. When decision-making resides with remote critical care specialists, mortality and length of stay are reduced [6].

The barriers to Tele-ICU adoption are not easily overcome. Cost is most often cited as the greatest hurdle, with start-up expenses often amounting to approximately $50,000-$100,000 per bed [1,3]. Rural hospitals can contract with urban centers for 24/7 access to clinicians in short-staffed specialties, but the financial strain induced by the pandemic led to the closure of 20 facilities in the first year in the United States and threatens many more [7]. Additionally, most regions require physicians to be licensed in the state they are practicing in, preventing remote practice in many cases [8]. Potential conflicts may arise in the acceptance of the remote provider’s medical decision by the in-house healthcare provider, as the autonomy of the staff with respect to patients varies according to the Tele-ICU model implemented [5]. The heterogeneity of the existing models of Tele-ICU has prevented the practical translation of research results into everyday practice, yielding mixed results with regard to the clinical benefits [9].

The COVID-19 pandemic has paved the way necessary for the adaptation of remote care as pressure placed by the pandemic on ICUs worldwide grew to an unparalleled level. The shortage of trained intensivists was further aggravated by the influx of critical patients, prompting facilities to search for creative ways to meet their needs [10]. Early and widespread utilization of telehealth modalities thankfully offered previously unrealized opportunities to study the various models and examine the results, hear the voices of those who adopted Tele-ICU to their workflow, and learn from their triumphs and failures.

## Review

Methods

Search Strategy

For this scoping review, we searched primary health research databases PubMed/MEDLINE and the Cumulative Index to Nursing and Allied Health Literature (CINAHL) for various ICU modalities employed during the COVID-19 pandemic to date. We searched peer-reviewed literature in the English language published from October 2020 through October 2021. We employed a Boolean search strategy, combining the following search terms: "Telemedicine” or “Virtual” or “Tele-ICU” or “digital health” or “Tele-intensive care” and “ICU” or “intensive care” and “COVID-19” or “pandemic.”

Inclusion and Exclusion Criteria and Outcome Measures

**Figure 1:**
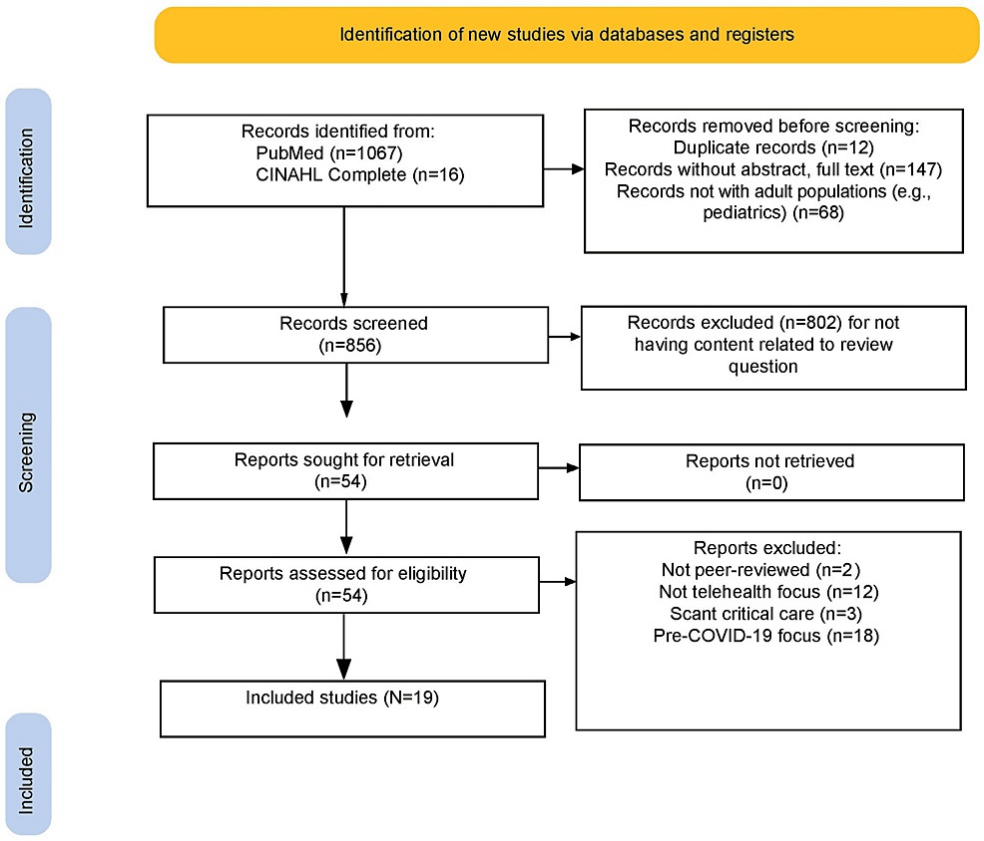
PRISMA flow diagram of the study selection PRISMA: Preferred Reporting Items for Systematic Reviews and Meta-Analyses: CINAHL: Cumulative Index to Nursing and Allied Health Literature

We restricted telemedicine to the critical care specialty with a physician-led focus, adult populations, removing any studies exclusive to nursing or mid-level providers. We cast a wide net for modalities, including all technological solutions whether in-house or remote delivery. To appropriately assess the utilization of new approaches to Tele-ICU and expansion of previously launched methods during the COVID-19 timelines, we excluded any studies where results were documented prior to November of 2019, in which a study may have been erroneously retrieved due to author mentions of the pandemic in light of their findings at the time of publication. Figure 1 illustrates the Preferred Reporting Items for Systematic Reviews and Meta-Analyses (PRISMA) flow chart of the study selection process.

Search

Our search yielded 1,067 records in PubMed and 16 results in CINAHL. We excluded all works that fell outside the pandemic timeline. After excluding duplicates and evaluating the articles for relevance, 19 articles were ultimately selected for this review (see Table 1). Studies included came from various countries, including the United States, Israel, the United Kingdom, Egypt, India, China, Brazil, Mexico, and Malaysia.

Data Extraction

After the application of inclusion criteria, the citation details (author, title, journal, year of publication) of relevant articles were entered into an Excel spreadsheet to evaluate the methods of each study, modalities utilized, variant reported results and limitations of individual studies. Articles were grouped by the primary focus of Tele-ICU delivery, whether teleconsultation, telemonitoring, telerounding, or family conferencing. Many contained overlapping themes, as every study included a central teleconsult component.

Results

Teleconsultation

Tele-ICU is not a concept novel to the COVID-19 pandemic, as its roots can be traced to the dawn of the millennium, offering solutions to critical care in underserved and rural communities. At the heart of remote critical care lies the concept of the intensivist consult, sometimes referred to as teleconsultation or telementoring. As noted in Table 1, each of these studies contains a component of teleconsultation.

Telemonitoring

Prior to the COVID-19 pandemic, remote monitoring was a primary Tele-ICU modality. Instead of expanding on this precedent, pandemic literature in this review devoted very little attention to the usefulness of remote monitoring and provided no evidence of its expansion.

Telerounding

Telerounding proved to be a useful teambuilding tool across the Tele-ICU model during COVID-19. Two studies found a great increase in the use of consultation with teleintensivists once regular remote rounding was instituted [11,12]. One of these studies incorporated education of residents via telerounding with intensivists [12].

Family Conferencing

Perhaps the most salient feature of Tele-ICU during the COVID-19 pandemic has been the use of technology to connect families otherwise separated by infection control procedures during end-of-life decision making and palliation. The Houston Methodist Hospital utilized registered nurses in the virtual ICU call center to connect families to the established “Consultant Bridge” platform used for teleconsults in order to make video calls to contribute to decision making and visitation [13]. Five studies demonstrated the use of teleconferencing equipment in place for consultations to connect otherwise sidelined family members with both the healthcare team and their loved ones [13-17].

Changing Infrastructure

Studies in Israel and London described newly launched hospital infrastructure, including construction in the former and rearranging in the latter, to accommodate green and red zones, insulating in-house intensivists from infection and utilizing their advanced training and experience to direct operations [14,18,19].

Discussion

The goal of this scoping review was to identify the available published data on implementing Tele-ICU modalities during the COVID-19 pandemic, highlighting unique and spontaneous solutions necessitated by the sudden and severe influx of critically ill patients.

Teleconsultation

The need for experienced physicians to manage rapidly declining critical patients was apparent in the early weeks of the COVID-19 pandemic, and hospital administrators quickly responded. Nearly half of all surveyed facilities in the United States either enlarged their ICU or were prepared to do so by using other units [20]. One nearly universal adoption involved the cancellation or postponement of elective surgeries (94.8%), according to a nationwide survey of hospitals conducted by Kerlin et al. [20]. The lack of inpatient census due to standard pre- and postoperative hospital stays freed the space and hands necessary to manage the burgeoning level of critical patients.

Studies in this review assess the transfer of crucial knowledge and experience from a relatively small pool of critical care physicians to lesser trained physicians and nurses to care for an expanded level of patients using best practices. This concept has made Tele-ICU known as a “force multiplier” in the critical care community with its hybrid use of remote personnel and digital health technologies [16,21]. Whether centralized through an intensivist “hub” or decentralized and generally in-house, an intensivist-led team comprised of those knowledgeable in intensive care consulted on an as-needed basis in real-time to assist the attending physician with ventilator settings, medication adjustments, and the interpretation of laboratory results and differential diagnoses.

In the Atrium Health study performed by Singh et al., virtual critical care augmentation was found to be more cost-effective than structural changes, though the initial investment in home workstations required extensive set-ups and support from their Information Technology department [22]. The Singh study found that teleintensivists worked well with triaging interfacility transfer decisions and resource utilization and noted that this was well received by physicians who had an intimate knowledge of hospital resources [22]. The role changes inherent in a hybrid virtual and in-person team initially created confusion, which was eventually overcome with frequent and intense communication strategies [22].

Case studies in Egypt demonstrated a role reversal for intensivists, with specialist confirmation of life-threatening circumstances in the context of COVID-19 infection requiring immediate intervention [23]. In the absence of an available cardiologist, the critical care physician sent a video echocardiogram so that a remote cardiologist could confirm cardiac tamponade, and the intensivist performed bedside echo-guided pericardiocentesis in response [23].

In 2018, the civilian and military experts within the United States Army began working on a model of centralized Tele-ICU after discussions with the Society of Critical Care Medicine (SCCM) annual congress, dubbing it the National Emergency Tele-Critical Care Network or NETCCN [16]. Led by Dr. Jeremy Pamplin of the Telemedicine and Advanced Technology Research Center (TATRC) of the United States Army Research and Development Command, the NETCCN project was deployed to develop programs that could be readily launched to accommodate the growing needs of the pandemic [16]. From the onset of COVID-19 through the end of 2020, four teams provided support to six rural hospitals with 248 remote experts [16]. NETCCN avoided interfacility transfers, provided many hours of shift coverage for local experts, and conducted multidisciplinary rounds via nearly 5,000 asynchronous and synchronous voice or video interactions [16].

A hospital in the Bronx, New York, during the surge, made use of the relative calm on the West Coast by calling upon intensivists in Washington State, as reported by Krouss et al. [11]. The relaxing of state licensure laws concerning physician practice during a declared state of emergency in New York enabled Washington state clinicians to care for patients remotely [11]. These volunteer critical care specialists recruited on the West Coast enabled Jacobi Medical Center to expand its ICU capabilities by 150% [11].

A Brazilian study described utilizing a common treatment and training protocol, by electing a central Respiratory ICU as the coordinating center for Tele-ICU for public hospitals in the state of São Paulo [17]. Their Information Technology department developed a platform entitled “iConf,” where all Tele-ICU data was stored using teleconsultations at prescheduled times on a daily basis [17]. Each session lasted for about an hour, giving approximately 10 minutes per patient for the remote physician to recommend diagnoses and discuss treatment options according to the established protocol [17]. During the study period, ICU mortality was reduced by 14.5% [17].

The University of California San Diego provided assistance to rural Imperial County and facilities in Mexico with a decentralized model of Tele-ICU for border hospitals [24]. A direct-care-oriented clinical service line assisted El Centro Regional Medical Center, while a conference-style educational model was utilized in Tijuana and Mexicali to work within the bounds of licensure [24]. In their decentralized model, teleintensivists provided consults from the comfort of their homes via mobile devices [24]. Of note, 78% of staff reported a boost in confidence levels in caring for COVID-19 patients in the ICU due to the launch of the platform [24].

Telemonitoring

Based on the pre-pandemic use of remote monitoring of vital signs for risk assessment, it was expected that this review would yield data on a wide expansion of telemonitoring, but it did not. The first and determined focus of providers in the early weeks of the pandemic was to find the most effective approach to managing critically ill COVID-19 patients, in the hope of reducing suffering and preserving life. The sharing of information between frontline physicians and those with significant critical care experience was valued over static monitoring of vital signs and risk assessment scores. There was a brief mention of it in the hybrid model of continuous monitoring and scheduled rounding employed in New York and Washington States [25]. This had been in place prior to the pandemic and was not enhanced or expanded.

Telerounding

Telerounding was found to improve teleconsultations in two studies [11,12]. The idea of including the Tele-ICU team during rounds did not occur intuitively at first, but when it became routine at a hospital in the Bronx, New York, consults rose from a mere 26 in the first 10 days to a total of 352 consults in the following 21 days [11]. In a post-study survey, 83.3% of staff found the integration of the teleintensivist into rounds was easy to manage [11]. Critical care physicians at St. Joseph Health in Washington used mobile carts with non-critical care physicians and residents at the bedside to round on 40-60 patients across three different hospital sites during their six-hour shift [25]. At the University of California San Diego Health System (UCSDHS), Ramnath et al. found that the time saved by virtual rounding allowed for expanded participation among providers with time constraints and limited flexibility [24].

Chinese specialists (ICU, respiratory, nephrology, endocrinology, infectious disease, cardiology, gastroenterology, and radiology) plugged into the nationwide Huayitong application for emergency consultations and telerounding [26]. Bedside physicians with less experience were able to deliver real-time data via videoconferencing. This study was unique in the composition of the Tele-ICU team as a multi-disciplinary team of specialists, rather than exclusively intensivists [26].

Telerounding for residents at the UCSDHS became a mainstay of education, as it was quickly incorporated into their daily activity [12]. The Team-based Remote E-learning and Tele-ICU program (TREAT) enabled a remote intensivist to guide treatment in areas without critical care physicians, while simultaneously teaching residents [12]. Beginning with three hours daily and 10 days monthly, it was rapidly expanded to four hours daily due to its popularity with local providers and patients within the first two weeks of inception [12].

In India, sharing of academic information between physicians after telerounds ensured that less experienced physicians were kept abreast of evolving treatment in the care of COVID-19 patients [27]. The model introduced by the Government of Karnataka included the involvement of social services, nursing, family support, and emotional and spiritual support of the patient through interdisciplinary involvement in rounds [27].

One neurology team in Malaysia utilized smart glasses technology to conduct virtual rounding in the neurological ICU [28]. Specialists monitored rounds via the glasses and directed residents daily through a review of physical examination parameters, medications, blood results, imaging, and wound management [28].

Family Conferencing

The pandemic ushered in an unusual era of restricted visitation for most hospitals. Tele-ICU remote communication had existed prior to COVID-19 but had been typically a supplement rather than a substitute for in-person conversations [15]. One survey found families perceived receiving less critical information such as updates via telehealth as favorable compared to more valuable discussions of treatment goals [15]. Ultimately, they found clinicians and families willing to adapt to the times but less likely to opt for teleconferencing under the more normative circumstances expected post-pandemic [15].

In a study by Dhala et al., they reported nurses in a call center assist families in connecting with their loved ones and the ICU team via telehealth modalities [13]. The platform known as the “Consultant Bridge” was already in place for teleconsultation and was easily adaptable to include family in virtual visitation and shared decision-making [13]. An average of nearly 22 calls were completed daily in April of 2020, and the visits increased over time until almost every COVID-19 patient had one visitor daily by using the platform [29]. Of the 230 family members who responded, over 86% expressed positive sentiments concerning the experience [29]. Of note, 44% of families found their loved one’s critical status or time taken for advanced procedures a great challenge to telehealth visitations [29]. In the U.S. government proof-of-concept study for NETCCN, the remote team utilized teleconferencing to support end-of-life care at a small hospital and even in a home situation with the family [16].

Changing Infrastructure

The most challenging adaptation by hospitals to COVID-19 in terms of time and expense was the modification of existing infrastructure to accommodate the influx of patients. A study in the UK reported hospitals tackling this with very little expense and no construction by clearing units and instituting a “green zone” and a contaminated “red zone”, preserving critical care physicians to manage more patients and reduce their infection risk [14]. Bedside computers were outfitted with pan-tilt-zoom cameras and the in-house Tele-ICU team managed three ICUs via tablets, while other non-critical care physicians directed patient care at the bedside [14]. Capacity soared from 18 ICU beds to 70, eventually supporting 27 ECMO beds rather than the baseline of four beds [14].

Sheba Medical Center in the Tel Aviv District of Israel utilized a space earmarked for an improvised war-time hospital by building a structure in an underground parking lot, accomodating the Tele-ICU team in a central control room with windows, enabling visualization of treatment directly [18]. This allowed the video to be replaced by low-tech communication such as hand-written notes and hand signals to overcome the acoustic barrier [18]. This is not to suggest that technology was sidelined, as staff utilized monitoring devices, video cameras, a robot, and a club car equipped to drive between beds for an overview of the entire unit [18]. The impromptu ICU was created with the central hub to prevent infection of personnel, supervise staff, reduce errors, enhance safety, increase collaboration, and manage operations effectively [19]. Pilosof et al. found improvement in patient safety measures through screen monitoring of rooms in the control area while line staff was caring for other patients [19]. Table 1 lays out a summary of the included studies.

**Table 1:** Summary of included studies

Study	Description	Setting	Outcomes
Rangappa et al., 2021: Telemonitoring and shared remote decision-making; state-wide protocols for COVID treatment [27]	Conducted telerounds for 28 districts over 10 weeks in April-June 2020	Bangalore, India, in-patient hospital critical care	Case fatality rate (CFR) of 1.17%. Bangalore hospitals not connected to Tele-ICU cared for 3,419 patients during the same period for a CFR of 2.69%
Scott et al., 2020: NETCCN (National Emergency Tele-Critical Care Network). Telemedicine and Advanced Technology Research Center (TATRC) [21]	Review of current technologies including telementoring, telemonitoring for isolation purposes using tablets, smart rings/bracelets for vital signs, and patient activity levels	Network to be nationwide in the United States	Proposal of a cloud-based low-resource stand-alone health information management system to create and coordinate virtual critical care wards
Macedo et al., 2021: ICUs equipped with telemedicine stations using dual high-resolution monitors, camera, echo-canceling microphone, and audio playback device [17]	Descriptive observational study of the implementation of Tele-ICU, followed by a retrospective analysis of clinical data of patients with COVID-19 admitted to ICUs between April and July of 2020	Division of Pulmonology, Institute of Coracao, Clinical Hospital, Faculty of Medicine at the University of Sao Paulo, Brazil, April-July 2020	ICU mortality decreased from 73.2% in April to 58.7% in July. ICU length of stay (LOS) decreased by one day and hospital LOS by five days
Krouss et al., 2020: Loosening of licensure restrictions, malpractice indemnification, volunteer critical care specialists, and securing video platforms for teleconsults [11]	Single-center, observational, QI initiative; 12-bed oncology floor converted to ICU. In-house critical care provided ICU consult. Allowed out-of-state physicians to practice on an emergency basis	Jacobi Medical Center, New York City, New York	62.5% of respondents agreed it was an overall positive experience; 68.8% felt it improved care and decreased anxiety. Initial consults were low, added structured rounding, and consults rose by a factor of six
Chandra et al., 2021: A hybrid model for Tele-ICU by cross-country telemonitoring and remote virtual rounding, connecting Washington physicians to New York [25]	To counteract the shortage of critical care physicians, collaboration with Washington State. A hybrid model developed for virtual rounding on critically ill, 40-60 patients per day	Northwell Health, 23 hospitals, 40 ICUs (500 beds) in downstate NY. Expanded to over 1,000 ICU beds during the COVID-19 surge. Tele-ICU expanded from 176 beds during pre-COVID to 450 beds	Northwell decreased staffing by 1.35 full-time employees for each six-hour shift. This freed up New York physicians who had been used outside of their specialty
Ramnath et al., 2021: Evaluated hybrid of in-person care and telemedicine [24]	Creation of a hybrid, multi-institutional critical care program. U.S.-based Tele-ICU had direct patient interaction, and Mexico Tele-ICU had a case-based model	University of California San Diego Health System and North Baja region of Mexico	An additional 19% of patients received evidence-based practice as a result of Tele-ICU; 78% of staff were more confident caring for COVID-19 patients in the ICU
Pamplin et al., 2021: U.S. Army Medical Research and Development Command tasked to develop research proposals to produce rapidly deployable modalities at the pandemic onset [16]	Health and Human Services Office of the Assistant Secretary for Preparedness and Response's need for rapidly deployable, hardware-light Tele-ICU to augment expertise when local experts were unavailable	Four NETCCN teams with 238 remote experts servicing six hospital locations with about 221 patients registered and nine ICU beds and 126 hospital beds	Experts assisted local providers with life-threatening cases and avoiding hospitalizations through remote home monitoring and supporting end-of-life care
Kennedy et al., 2021: Review of telephone and video communication [15]	A qualitative interviewing study was conducted with a sample of 21 family members and 14 clinicians within the medical center. Content analysis was used to develop a codebook for interview transcripts	University of Pittsburg School of Medicine/UPMC Presbyterian Hospital	Phone and video communication were somewhat effective but not as intimate as in-person communication. Phone calls were useful for brief updates, and video calls were preferable for decisions
Sasangohar et al., 2021: Evaluated attitudes toward family visitation via telemedicine methods for ICU patients during COVID [29]	Interview of 346 family members of ICU patients post-visit via phone	Houston Methodist Hospital, Texas	>86% positive sentiments. Concerns: inability to communicate due to patient status (44%); technical difficulties (35%); lack of touch and physical presence (11%)
Li et al., 2021: Evaluated remote CT analysis and teleconsultation for medical personnel untrained in critical care. Mobile app established [26]	Case study: Telemedicine mobile application Huayitong developed as a new platform for patients to access telehealth consultations. Provided triage, rounds, education, and consultations	West China Hospital of Sichuan University and remote services to 660 collaborating hospitals, in 183 cities around Western China	Huayitong was installed on smart devices. 10,557 online COVID-19 consultations were conducted for 6,662 patients. Quality of care improved at subordinate hospitals; improved health outcomes in rural areas
Dhala et al., 2020: Evaluation of the implementation of virtual ICU at Houston Methodist Hospital [13]	Case study: March 2020; virtual ICUs expanded to include night coverage for specialty ICUs. By April, implementing virtual care during the day due to shortages owing to ill staff	Houston Methodist ICU at the onset of the COVID-19 pandemic	20 to 40 calls per day for family visitation through the virtual ICU system; surveys showed approval. Family members engaged in decisions and palliation
Kerlin et al., 2021: U.S. national hospital survey of COVID-19 strategies [20]	A cross-sectional survey of U.S. hospitals with ICUs stratified based on size, teaching status, health system membership, and incidence of COVID-19 in geographic regions and the greatest number of cases selected	U.S. survey of chief nursing officers from 169 nationwide hospitals	Actions to increase or preserve ICU staff, including the use of ICU telemedicine, were highly variable, without a single dominant strategy. Expanded existing Tele-ICU programs by 39.1%, significant minority introduced new programs (25.6%)
Mohammed Sheata et al., 2021: Expert consultation in the emergency setting, such as interpretation of echocardiogram by a cardiologist to confirm cardiac tamponade [23]	Case studies: Described how telemedicine played a vital role in the management of two patients with cardiac emergencies associated with COVID-19	Bedside ICU at Ain Shams University Hospital, Cairo, Egypt with associated COVID-19 infections and cardiopulmonary emergencies	Lifesaving interventions in pulmonary embolus and cardiac tamponade in the setting of COVID-19 were initiated with confirmation of diagnosis via telemedicine to cardiology from intensivists at the bedside
Pilosof et al., 2021: Newly constructed ICU with in-house consultation via segregated intensivists [18]	Semi-structured staff interviews. Field observations of COVID-19 ICU were conducted for qualitative analysis. Additional data included media coverage, hospital webinars, and analysis of architectural plans	Sheba Medical Center, Ramat Gan, Israel (Tel Aviv). 1,900-bed tertiary hospital, March-August 2020	A hybrid model of virtual and physical visibility to overcome challenges associated with telemedicine technologies. Variety of tools; architectural design, InTouch Telepresence robot, and club car to maximize access
Pilosof et al., 2021: Central control booth design of in-house Tele-ICU [19]	40 formal interviews with staff, and four days of field observations at the COVID ICU and IMU	Sheba Medical Center, Ramat Gan, Israel (Tel Aviv). 1,900-bed tertiary hospital, March-August 2020	Introduction of new ways to supervise and monitor to decrease errors. Promotes staff safety by minimizing contact with the virus
Munusamy et al., 2021: Residents and specialists conducted consecutive virtual and physical ward rounds on neurocritical patients [28]	Virtual ward rounds were conducted by specialists receiving audiovisual information from residents wearing smart glasses. Management plans of both rounds compared; intra-rater reliability measured	Centre for Biomedical and Technology Integration, Kuala Lumpur, Malaysia. Installed and integrated a secure mobile telemedicine system - MEDCOM Vision	Ten paired ward rounds were performed on 103 neurocritical care patients with excellent overall intra-rater reliability. Qualitative analysis indicated wide user acceptance and a high satisfaction rate
Singh et al., 2020: Evaluated equipping non-ICU rooms with virtual critical care interface technology [22]	130 mobile health carts for ICU and ED. Remote workstations for physicians to work from home when quarantined	Atrium Health, >40 hospitals and 900 care locations in Carolinas and Georgia. Virtual Critical Care (VCC) served >300 adult critical care beds across 12 hospitals pre-COVID-19	Expansion of tele-critical care increased ICU beds. Virtual physicians had some difficulty in communication with staff at the bedside due to PPE restriction of audiovisual communication
Leverone et al., 2020: Exploration of remote critical care education and consultation in remote areas without intensivist expertise [12]	Team-based Remote E-Learning and Tele-ICU (TREAT) used experienced critical care physicians to provide remote guidance for areas without intensivists while training residents	University of California San Diego Medical Center	62% of clinicians cited access to expertise regarding mechanical ventilation as most important, and 19% the ability to ask questions to a critical care expert
Igra et al., 2020: Videoconferencing in-house to connect intensivists with inexperienced physicians caring for critical patients in newly opened makeshift ICUs [14]	Case studies: Impromptu ICUs were formed by opening contiguous units, staffing with non-critical care personnel, and using bedside cameras to connect with intensivists	The Royal Brompton & Harefield NHS Foundation Trust combines two west-London hospitals providing tertiary cardiothoracic care, including critical care with extracorporeal membrane oxygenation (ECMO) support	Allowed for live video feeds, hands-free at the bedside for relatively low cost and fast set-up. Important implications for disaster response and future pandemics as a model for expanding hospitals without utilizing outside agencies

Limitations

There were many new approaches to COVID-19 in hospital systems worldwide not studied scientifically or published. This sample does not provide an adequate assessment of all ideas generated for Tele-ICU through the course of the pandemic, rather those studied, analyzed, and subjected to peer review.

We did not analyze the element of cost in this review, as only a few studies mentioned it and it is difficult to extrapolate useful information during a state of emergency where donations of equipment are plentiful and volunteer staff easy to find. Low-cost solutions were introduced with equipment on hand, though these plans were intended to be temporary and long-term expenses would need to be considered further, as cost is the primary concern for any hospital administration planning to launch a Tele-ICU program.

Future research

Further research to gather more information on the cost of Tele-ICU innovations in a post-pandemic time is needed. Realistic staffing and equipment that can stand the test of time must be put in place, and start-up costs and an annual budget to maintain the program must be given careful consideration. This review touched on the waiver of billing by teleintensivists to prevent delays in additional training time. The viability of billing insurances for Tele-ICU services to sustain these units should be investigated.

Post-pandemic examination of cross-state licensure limitations should also be considered, as these restrictions and other liabilities were relaxed in many instances to manage the crisis. Future research should explore the roadblocks to more centralized, remote, and standardized Tele-ICU models, such as NETCCN. An examination of the most reliable modes of telecommunication for information exchange, the practical composition of the Tele-ICU team, and standardized access to electronic health records would also be of great value.

## Conclusions

While Tele-ICU is not a concept novel to the COVID-19 pandemic, it has been expanded and deployed creatively as the need for training and experience has grown with the emergence of COVID-19. A shortage of critical care staff relative to the surge of critically ill patients drove hospitals to look for new ways to protect these physicians and nurses from infection and share their knowledge in-house, across hospital systems, and even globally. The advanced familiarity and comfort with telehealth for clinicians, patients, and family members are expected to endure and grow further from here, paving the way for the broader acceptance and expansion of the Tele-ICU concept.
